# Morphological characteristics of retinal vessels in eyes with high myopia: Ultra-wide field images analyzed by artificial intelligence using a transfer learning system

**DOI:** 10.3389/fmed.2022.956179

**Published:** 2023-02-16

**Authors:** Jianbo Mao, Xinyi Deng, Yu Ye, Hui Liu, Yuyan Fang, Zhengxi Zhang, Nuo Chen, Mingzhai Sun, Lijun Shen

**Affiliations:** ^1^Department of Ophthalmology, Center for Rehabilitation Medicine, Zhejiang Provincial People’s Hospital (Affiliated People’s Hospital, Hangzhou Medical College), Hangzhou, Zhejiang, China; ^2^Eye Hospital of Wenzhou Medical University, Wenzhou, Zhejiang, China; ^3^Department of Precision Machinery and Instrumentation, University of Science and Technology of China, Hefei, China

**Keywords:** high myopia, ultra-wide field imaging, deep learning, vascular morphology, choroidal neovascularization

## Abstract

**Purpose:**

The purpose of this study is to investigate the retinal vascular morphological characteristics in high myopia patients of different severity.

**Methods:**

317 eyes of high myopia patients and 104 eyes of healthy control subjects were included in this study. The severity of high myopia patients is classified into C0–C4 according to the Meta Analysis of the Pathologic Myopia (META-PM) classification and their vascular morphological characteristics in ultra-wide field imaging were analyzed using transfer learning methods and RU-net. Correlation with axial length (AL), best corrected visual acuity (BCVA) and age was analyzed. In addition, the vascular morphological characteristics of myopic choroidal neovascularization (mCNV) patients and their matched high myopia patients were compared.

**Results:**

The RU-net and transfer learning system of blood vessel segmentation had an accuracy of 98.24%, a sensitivity of 71.42%, a specificity of 99.37%, a precision of 73.68% and a F1 score of 72.29. Compared with healthy control group, high myopia group had smaller vessel angle (31.12 ± 2.27 vs. 32.33 ± 2.14), smaller fractal dimension (Df) (1.383 ± 0.060 vs. 1.424 ± 0.038), smaller vessel density (2.57 ± 0.96 vs. 3.92 ± 0.93) and fewer vascular branches (201.87 ± 75.92 vs. 271.31 ± 67.37), all *P* < 0.001. With the increase of myopia maculopathy severity, vessel angle, Df, vessel density and vascular branches significantly decreased (all *P* < 0.001). There were significant correlations of these characteristics with AL, BCVA and age. Patients with mCNV tended to have larger vessel density (*P* < 0.001) and more vascular branches (*P* = 0.045).

**Conclusion:**

The RU-net and transfer learning technology used in this study has an accuracy of 98.24%, thus has good performance in quantitative analysis of vascular morphological characteristics in Ultra-wide field images. Along with the increase of myopic maculopathy severity and the elongation of eyeball, vessel angle, Df, vessel density and vascular branches decreased. Myopic CNV patients have larger vessel density and more vascular branches.

## 1. Introduction

Globally, myopia is one of the most common eye diseases. High myopia (HM) is associated with a spherical equivalent greater than –6.00 diopters (D) or with an eyeball axial length (AL) longer than 26 mm (1). In the last 10–15 years, the prevalence of HM has increased from less than 10–20% in parts of Asia, such as Korea, Japan, and several parts of China. It is predicted that 9.8% of the world population will have HM by 2050 (2). HM may lead to myopic maculopathy and greatly increase the risk of blindness. The elongation of the eyeball is the predominant mechanism in the progression of myopic maculopathy, which is associated with significant retinal vessel morphologic alterations. Therefore, investigating the morphological features of retinal vessels may provide critical clues for understanding the pathophysiology of different stages of HM-related maculopathy.

Different methods have been proposed for the grading of HM maculopathy. Avila et al. (3) suggested a grading system in 1984. However, controversy lies in the posterior staphyloma in M2 and lacquer cracks in M3. Ruiz-Medrano et al. (4) developed a comprehensive but rather complex grading system based on retinal atrophy, traction, and neovascularization. Focusing on the degenerative changes in HM, we adopted the International Photographic Classification and Grading System for Myopic Maculopathy, which is effective and widely recognized in clinics. This system was proposed by Ohno-Matsui et al. (5) based upon the meta-analysis of pathologic myopia (META-PM). In this grading scheme, myopic maculopathy is classified into 5 grades follows: no myopic retinal degenerative lesion (C0), tessellated fundus (C1), diffuse chorioretinal atrophy (C2), patchy chorioretinal atrophy (C3), macular atrophy (C4), and myopic choroidal neovascularization (mCNV) as one of the plus signs. Vascular findings based on optical coherence tomography (OCT) and OCT angiopathy (OCTA) (6–10) are highly variable, e.g., decreased macular microvascular density in both the superficial and deep vascular plexuses, increased size of the foveal avascular zone, or even the absence of change in different the stages of HM eyes. In fundus photographs, the area of parapapillary atrophy in different stages of HM has been measured (11), and decreases in the caliber of the retinal vessels have been demonstrated (12). Few studies have used a panoramic perspective to investigate retinal vessel morphology in cases of HM maculopathy. Ultra-wide field (UWF) imaging is a rapidly evolving diagnostic modality that can capture the peripheral retina in a single image (13), thus providing comprehensive retinal imaging.

Retinal vascular morphological characteristics altered in a variety of retinal diseases, such as diabetic retinopathy and retinal vein occlusion, which often combined with retinal vascular tortuosity and dilatation. Therefore, quantitative vascular analysis is conductive to diagnose the disease and determine the severity. Our previous study (14) suggested smaller vascular angle and vessel density in familial exudative vitreoretinopathy in UWF images, provides great clinical value for the screening and diagnosing of these rare diseases. However, no prior studies have yet been performed to analyze quantitative vascular morphological changes in HM and mCNV in UWF images.

In recent years, deep learning methods have been widely applied to ophthalmic diseases, e.g., diabetic retinopathy (15), glaucoma (16), and age-related macular degeneration (17). In our previous study, we used deep learning to automatically achieve vascular segmentation in retinopathy of prematurity (18) and in familial exudative vitreoretinopathy (14). In this study, we applied the deep learning and transfer learning scheme to segment the vessels in UWF images of HM. We then investigated variations of vascular morphological characteristics at the different degrees in C0–C4. We also analyzed the vascular characteristics of mCNV. Hoping to further understanding the progression of HM and the changes in the vasculature of HM as the severity of the disease increases, and provide new insights to study the pathological mechanisms of mCNV.

## 2. Material and methods

### 2.1. Subjects

HM cases (203 subjects, 317 eyes) and healthy control (HC) (61 subjects, 104 eyes) were recruited from July 2020 to January 2021 in Eye Hospital of Wenzhou Medical University. Each subject underwent comprehensive ocular examination including subjective refraction for best corrected visual acuity (BCVA), fundus photography (Topcon 50DX, Topcon, Tokyo, Japan) for META-PM grading, UWF retinal imaging (Optos 200Tx Imaging System, Optos PLC, Dunfermline, Scotland, UK), and AL determination (IOL Master, Carl Zeiss Meditec, Jena, Germany).

Inclusion criteria were age ≥18 years old, AL ≥ 26 mm, or spherical equivalent < –6.00. Exclusion criteria were ocular trauma; vitreoretinal or systemic disease that could affect the eyes, such as diabetes mellitus and hypertension; or significant refractive media opacity that could affect the image quality.

### 2.2. UWF retinal imaging examination

All HM patients and HC subjects underwent UWF retinal imaging with dilated pupils, including green laser images (wavelength 532 nm), red laser images (wavelength 633 nm) and pseudocolor (two-color) images. In this study, we collected the green laser images because of the high contrast between the vasculature and the retinal background. Then the vascular characteristics was analyzed including vessel angle, fractal dimension (Df), vascular density, and vascular branches. Images from 317 eyes of the 203 HM patients and 92 eyes from the 49 HC subjects were included.

### 2.3. Myopic maculopathy grading

According to the META-PM grading system, we classified all images into one of the five degrees, i.e., C0–C4, as proposed by Ohno-Matsui et al. (5). In addition, we identified CNVs through OCTA scans (Optovue, Inc., Fremont, CA, USA). The work of grading was performed by two of the co-authors (JM and XD). In cases where there was disagreement, the final decision was made by a senior specialized ophthalmologist (corresponding author LS).

### 2.4. Analysis of vascular characteristic by artificial intelligence

**FIGURE 1:**
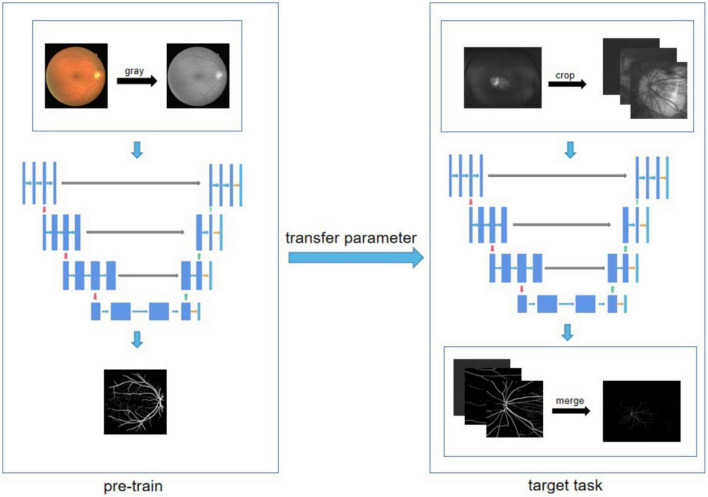
The schematic diagram of the transfer learning technology used in this study.

Transfer learning technology was used to analyze the characteristic of retinal vessels as shown in Figure 1. The reinforced U-net (RU-net) is one of the branches of deep learning, achieves very good performance on biomedical segmentations with small number of training images (19). We first used 380 regular fundus images from public datasets to pre-train on the RU-net network (20, 21). The pre-trained RU-net network had a feature extraction ability for blood vessels and a blood vessel segmentation effect. After that, the pre-trained RU-net network was trained again on the annotated wide-angle fundus images. In the next step, 50 manually-labeled UWF fundus images were used to retrain the Ru-net network. In the data preprocessing stage, each UWF fundus image was cropped to 576 × 576 pixels. The output slices were then combined to create the final output model composed of 3,900 × 3,072 pixels.

Based on the pathological features of HM, we evaluated the vessel angle, fractal dimension (Df), vascular density, and number of vascular branches. In the process of angle measurement, we connected the center of the macula and optic disc as the reference axis and calculated the angle between all vessels and that axis. Df was determined using the standard box-counting method proposed by Stosic and Mainster (22, 23). The vascular density was evaluated by calculating the ratio of blood vessel points to the entire area of the map after removing the avascular area around the binary vessel images. The number of vascular branches was detected by counting the number of pixels in a connected domain that broke the skeletonized blood vessels into branches. The specific analysis methods were detailed in our previous article (14).

### 2.5. Statistical analysis

Statistical analysis was performed using SPSS software (version 26.0, SPSS Inc., Chicago, IL, USA). All data were expressed as means ± standard deviations. The demographic and clinical characteristics were compared between the groups using the chi-square test or one-way analysis of variance. Associations between AL, BCVA, age, and vascular characteristics among each group were assessed using Pearson correlation tests. Paired t-tests were used to analyze paired samples. *P*-values < 0.05 were considered to be statistically significant.

## 3. Results

### 3.1. Retinal vascular segmentation in UWF images

**FIGURE 2:**
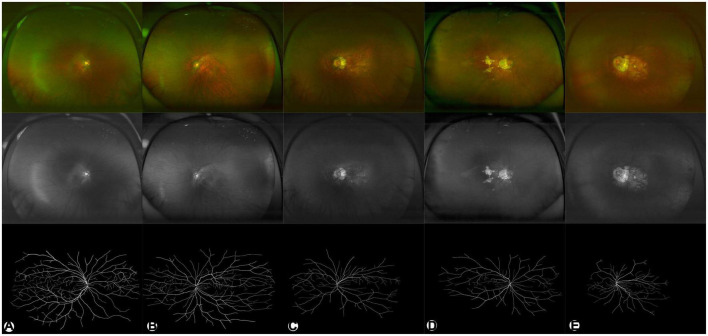
Representative examples of HM in different degrees. **(A–E)** are C0–C4, respectively. The upper panel (in color) shows pseudocolor images. The middle panel in gray shows green laser images. The lower panel in black/white shows segmented images of binarized skeletonized blood vessels.

The performance of the system used in this study was evaluated using five metrics, including accuracy, sensitivity, specificity, precision and F1 score. The results showed that the RU-net combined with transfer learning achieved 98.24% accuracy, 71.42% sensitivity, 99.37% specificity, 73.68% precision and 72.29 F1 score in retinal vascular segmentation. Representative examples of skeletonized blood vessels of HM in different maculopathy stages were shown in Figure 2.

### 3.2. Participant demographics

The ages of the HC subjects ranged from 20 to 84 years, with a mean of 43.8 ± 15.6 years. The ages of HM patients ranged from 18 to 85 years, with a mean of 46.2 ± 16.7 years. The male-to-female ratios of the HC and HM groups were 0.73 (44/60) and 0.50 (105/212) respectively. The age and gender were comparable between the two groups (*P* = 0.203, 0.089, respectively).

### 3.3. Retinal vascular morphology in HC and HM group

**FIGURE 3:**
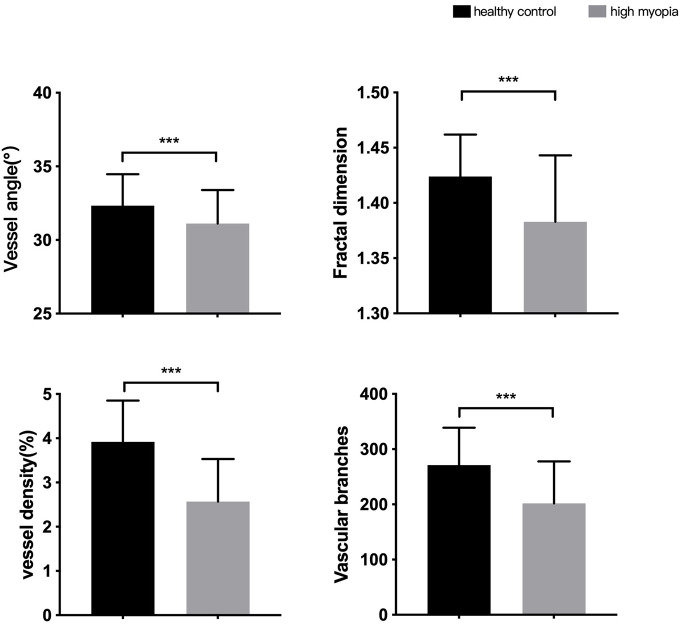
Differences in morphological characteristics between HM group and healthy control group. ***P < 0.001.

Eyes in the HM group had smaller vessel angles (31.12 ± 2.27 vs. 32.33 ± 2.14), smaller Df values (1.383 ± 0.060 vs. 1.424 ± 0.038), less vessel density (2.57 ± 0.96 vs. 3.92 ± 0.93) and less vascular branches (201.87 ± 75.92 vs. 271.31 ± 67.37) than in the HC group (all *P* < 0.001, Figure 3).

### 3.4. Retinal vascular morphology of different degrees in HM group

There were significant intergroup differences of age, BCVA, AL, and vascular characteristics, including vessel angle, Df, vessel density, and the number of vascular branches (all *P* < 0.001, Table 1). With the increase in severity of myopic maculopathy, age tended to be older, BCVA tended to be worse, and AL tended to be longer. As the disease progressed, vessel angles, Dfs, and vessel densities were smaller, and there were fewer vascular branches. In pairwise comparisons (Table 2), there were many significant differences in the measured parameters as the disease progressed from C0 through C4; however, some parameters did not change significantly in the transition from one stage to the next.

**TABLE 1:** Demographic, morphological, and vascular characteristics of the five HM group.

Characteristics	C0	C1	C2	C3	C4	*P*
No. of subjects	31	82	108	49	46	–
Gender (male/female)	13/18	28/54	42/67	8/41	14/32	0.062
Age (years)	29.0 ± 8.2	33.3 ± 11.6	47.7 ± 12.7	56.7 ± 11.6	66.0 ± 10.6	<0.001
BCVA (logMAR)	0.04 ± 0.07	0.09 ± 0.16	0.30 ± 0.35	0.69 ± 0.59	1.38 ± 0.91	<0.001
AL (mm)	26.47 ± 0.95	27.27 ± 1.09	28.86 ± 1.87	30.44 ± 2.11	30.06 ± 2.05	<0.001
Vessel angle (°)	32.55 ± 1.87	31.87 ± 2.24	30.72 ± 2.11	30.47 ± 1.96	30.43 ± 2.51	<0.001
Df	1.45 ± 0.03	1.43 ± 0.03	1.38 ± 0.04	1.36 ± 0.04	1.30 ± 0.04	<0.001
Vessel density (%)	3.70 ± 0.69	3.12 ± 0.72	2.34 ± 0.74	2.11 ± 0.71	1.83 ± 0.99	<0.001
Number of vascular branches	290.06 ± 58.80	250.76 ± 64.20	189.30 ± 59.06	163.67 ± 40.42	124.70 ± 52.59	<0.001

BCVA, best corrected visual acuity presented as the log (minimum angle of resolution); AL, axial length; Df, fractal dimension.

**TABLE 2:** Pairwise comparisons of morphological characteristics among HM groups.

	Vessel angle					Df			
**Grade**	**C0**	**C1**	**C2**	**C3**	**Grade**	**C0**	**C1**	**C2**	**C3**
C1	0.138				C1	0.035			
C2	<0.001	<0.001			C2	<0.001	<0.001		
C3	<0.001	<0.001	0.489		C3	<0.001	<0.001	0.013	
C4	<0.001	<0.001	0.439	0.937	C4	<0.001	<0.001	<0.001	<0.001
	**Vessel density**					**Vascular branches**			
**Grade**	**C0**	**C1**	**C2**	**C3**	**Grade**	**C0**	**C1**	**C2**	**C3**
C1	0.002				C1	0.002			
C2	<0.001	<0.001			C2	<0.001	<0.001		
C3	<0.001	<0.001	0.344		C3	<0.001	<0.001	0.014	
C4	<0.001	<0.001	0.019	0.499	C4	<0.001	<0.001	<0.001	0.002

C0, no myopic retinal degenerative lesion; C1, tessellated fundus; C2, diffuse chorioretinal atrophy; C3, patchy chorioretinal atrophy; C4, macular atrophy.

### 3.5. Correlations of AL, BCVA, and age with retinal vascular morphology

AL, BCVA, and age were each negatively correlated with vessel angle, Df, vessel density, and vascular branching (Pearson correlation analysis, all *P* < 0.05, Table 3).

**TABLE 3:** Correlations of morphological characteristics with AL, BCVA, and age.

Characteristics	AL		BCVA		Age	
	***r***	***P***	***r***	***P***	***r***	***P***
Vessel angle(°)	–0.156	0.008	–0.223	<0.001	–0.142	0.012
Df	-0.489	<0.001	–0.556	<0.001	–0.705	<0.001
Vessel density (%)	–0.505	<0.001	–0.355	<0.001	–0.566	<0.001
Vascular branches	–0.430	<0.001	–0.481	<0.001	–0.669	<0.001

AL, axial length; BCVA, best corrected visual acuity; Df, fractal dimension.

### 3.6. Comparison between groups with and without CNV

We selected 30 HM patients and 30 mCNV patients, who were matched for age, sex, AL, and myopic maculopathy grade, and compared the vascular characteristics between the two groups. Each group had 1 eye of C0, 16 eyes of C2, 10 eyes of C3, and 3 eyes of C4. There were no significant differences between HM patients with or without CNVs with regard to male/female ratio, age, BCVA, AL, vessel angle, or Df (Table 4). However, the vessel density for those without CNV, 2.03 ± 0.64%, was smaller than for those with CNV, 2.65 ± 0.87% (*P* < 0.001, Table 4). In contrast, the number of vascular branches for those without CNV, 163.43 ± 55.89, was fewer than for those with it, 187.37 ± 61.08 (*P* = 0.045).

**TABLE 4:** Demographic and morphological characteristics of HM patients with and without CNV.

Characteristics	With CNV	Without CNV	*P*
Gender (male/female)	17/13	17/13	1.000
Age (years)	53.1 ± 14.8	53.50 ± 12.768	0.600
BCVA	0.53 ± 0.43	0.61 ± 0.67	0.429
AL (mm)	28.75 ± 1.44	29.04 ± 1.74	0.442
Vessel angle (°)	30.71 ± 1.63	30.39 ± 2.16	0.510
Df	1.362 ± 0.044	1.356 ± 0.051	0.593
Vessel density (%)	2.65 ± 0.87	2.03 ± 0.64	<0.001
Vascular branches	187.37 ± 61.08	163.43 ± 55.89	0.045

BCVA, best corrected visual acuity; AL, axial length; Df, fractal dimension; CNV, choroidal neovascularization.

## 4. Discussion

Combining UWF retinal imaging and deep learning technology, the present study investigated and quantified retinal vascular morphology parameters in HM based on a comprehensive perspective of the retina. We found that retinal vessel angle, Df, vessel density, and the amount of vascular branching all decreased in association with increased severity of myopic maculopathy and eyeball elongation. Each of these changes were correlated with age, AL, and BCVA. In the same severity grade, the increase of vessel density and the decrease of vascular branching were risk factors of CNV in HM patients.

Our HM patients had significantly smaller vessel angles, Dfs, vessel density and fewer vascular branches than did in the healthy individuals. These findings were in line with previous studies by other authors using different methods. For instance, Che Azemin et al. (24) found that younger myopic subjects had smaller retinal vascular Dfs, indicating less complexity, than in age-comparable emmetropic subjects. Similarly, Li et al. (25) also demonstrated the apparent reduction of Df in HM subjects, which was associated with longer AL. Using laser Doppler velocimetry, Shimada et al. (26) quantified decreased retinal blood flow in highly myopic eyes.

The vascular morphological changed with increased myopic maculopathy indicate ongoing atrophic changes. Thus, our results were consistent with those reported by others. Based on color fundus photographs of HM patients, Jonas et al. (27) suggested that the angle kappa between the temporal superior and temporal inferior arterial arcade decreased with longer AL, and this change was correlated with larger atrophic lesions. Li et al. reported that fundus autofluorescence in patients with different grades of META-PM–classified myopic maculopathy was correlated with age, BCVA, AL, and subfoveal choroidal thickness (28). Zhao et al. (29) showed that AL was significantly longer in association with the severity of myopic maculopathy from C0 to C3, but not significantly different between C3 and C4. We also found no significant differences in AL, vessel angle or vessel density between C3 and C4. Collectively, these studies suggest that there may not be significant progressive changes between C3 and C4.

The vascular morphological changes were related with age, AL, and BCVA. Pathological myopia is characterized by degeneration of chorioretinal structure and vasculature, and results in vision impairment (9). With the increasing of age, AL would elongate, spherical equivalent would increase and the vascular morphology would change accordingly. It has been proven by past research that the increase in severity of HM maculopathy has a strong association with older age (2), which is consistent with our results. Khan et al. (30) and Guo et al. (31) investigated the morphology by OCTA of the superficial retinal capillary plexus over a 3 × 3 mm^2^ fovea-centered area and found a significant inverse association between AL and vascular density. Likewise, study of mild, moderate, high and extreme myopia patients in 6 × 6 mm^2^ OCTA showed the decrease of vascular density and negative correlations between Df and AL (32). The author suggested that AL impact retinal vascular density than other parameters since axial elongation contributes more to the structural alterations in the posterior segment. In regards to the kappa angle between the temporal superior and inferior arterial arcade, studies were consistent that kappa angle was correlated to myopia progression and AL elongation, which was in line with our study (33, 34).

In this study, when the age and severity grade are comparable, mCNV patients tended to have larger vessel density and more vascular branches. Ren et al. (35) suggested that compared with simple hemorrhage, myopic CNVs had much more severe atrophic lesions in thinner choroids, which can lead to insufficient blood supply and consequently induce the development of CNV. Similarly, Xie et al. (36) demonstrated that a relatively thinner choroid is a biological indicator for myopic CNV presence or development. Therefore, for such cases, close follow-up, early diagnosis, and prompt therapy are necessary.

We applied transfer learning technology to automatically analyze the morphological features of the retinal vessels. Due to the difficulty of manually annotating images, there was insufficient training data available to utilize deep learning technology in the annotation of the UWF images. However, more training data are available for annotated regular fundus images. Therefore, we used transfer learning to solve the problem of insufficient training data for the UWF images. Regular fundus cameras usually have a field of view of 45°, while our UWF imaging system has a 200° panoramic field of view. Utilizing the high resolution of the UWF imaging in the stage of data preprocessing, we segmented each UWF image, dividing the 3,900 × 3,072 pixels into images composed of 576 × 576 pixels slices. This method not only solves the memory overflow problem caused during the training process, but also increases the training data of the network, so that the network has an improved blood vessel segmentation ability. The transfer learning method used in this study solves the problem with the lack of sufficient training data, and the training network is more accurate in the blood vessel segmentation of the UWF fundus images.

Our study has several limitations. First, this was a single-center, hospital-based study. Second, the peripheral deformation due to ultra-wide photography is difficult to correct. Moreover, the sample size of the mCNV group was relatively small; thus, the results should be considered cautiously. Further studies may eliminate these problems through multicenter testing to enlarge the sample size and through ongoing advances in artificial intelligence and deep learning methods.

In conclusion, high myopia patients had smaller vessel angles, Dfs, vessel density and fewer vascular branches. As the severity of myopic maculopathy and AL increased, vessel angle, Dfs, vessel density and vascular branches decreased. Understanding these morphological variations may provide hints about the high myopia pathological process.

## Data availability statement

The raw data supporting the conclusions of this article will be made available by the authors, without undue reservation.

## Ethics statement

All procedures performed in this study involving human participants were in accordance with the ethical standards of the Institutional and the National Research Committee and with the 1964 Helsinki Declaration and its later amendments or comparable ethical standards. The study was approved by the Wenzhou Medical University Affiliated Eye Hospital Ethics Committee. All patients provided written informed consent for the inclusion in the study.

## Author contributions

JM, LS, and MS contributed to the conception and design of the study. XD collected the images and wrote the first draft of the manuscript. YY and HL analyzed the images and performed the statistical analysis. YF, ZZ, and NC wrote sections of the manuscript. All authors contributed to the manuscript revision, read, and approved the submitted version.
